# Structural basis for recognition of Rift Valley fever virus Gn protein by a human neutralizing monoclonal antibody with a kappa light chain

**DOI:** 10.1371/journal.ppat.1013926

**Published:** 2026-02-17

**Authors:** Guido C. Paesen, Nathaniel S. Chapman, Jonna B. Westover, Cynthia M. McMillen, Natalia A. Kuzmina, Emmett A. Dews, Luke Myers, Robert Stass, Joel M. Montgomery, Alexander Bukreyev, Amy L. Hartman, Brian B. Gowen, James E. Crowe, Thomas A. Bowden

**Affiliations:** 1 Division of Structural Biology, Centre for Human Genetics, University of Oxford, Oxford, United Kingdom; 2 Department of Pathology, Microbiology, and Immunology, Vanderbilt University Medical Center, Nashville, Tennessee, United States of America; 3 Vanderbilt Center for Antibody Therapeutics, Vanderbilt University Medical Center, Nashville, Tennessee, United States of America; 4 Department of Animal, Dairy and Veterinary Sciences, Utah State University, Logan, Utah, United States of America; 5 University of Pittsburgh, Center for Vaccine Research, Pittsburgh, Pennsylvania, United States of America; 6 University of Pittsburgh, Department of Infectious Diseases and Microbiology, School of Public Health, Pittsburgh, Pennsylvania, United States of America; 7 Department of Pathology, University of Texas Medical Branch, Galveston, Texas, United States of America; 8 Galveston National Laboratory, Galveston, Texas, United States of America; 9 Department of Microbiology and Immunology, University of Texas Medical Branch, Galveston, Texas, United States of America; 10 Viral Special Pathogens Branch, Division of High-Consequence Pathogens and Pathology, Centers for Disease Control and Prevention, Atlanta, Georgia, United States of America; 11 Department of Pediatrics, Vanderbilt University Medical Center, Nashville, Tennessee, United States of America; Icahn School of Medicine at Mount Sinai, UNITED STATES OF AMERICA

## Abstract

Rift Valley fever virus (RVFV) poses a continued threat to human health and animal husbandry. Two neutralizing and protective human monoclonal antibodies (mAbs), RVFV-268 and RVFV-379, exhibit similar affinities and epitope footprints on the Gn glycoprotein component of the RVFV Gn-Gc capsomeric lattice. Here, we define fine details of the biophysical determinants of Gn recognition used by RVFV human monoclonal antibodies through studying an antibody encoded by a set of recombined genes not previously identified in RVFV antibodies. We find that RVFV-379 exhibits a larger footprint than that observed for RVFV-268 and other antibodies targeting the same region, which involves major contributions of both the light and heavy chains. RVFV-379 also uses an oblique angle of approach towards the virion surface that contrasts with the perpendicular angle of engagement observed for some other potently neutralizing human mAbs. Further, consistent with amino acid sequence variation within and proximal to the RVFV-379 epitope, *in vitro* neutralization screening reveals a limited degree of neutralization breadth across prevalent RVFV strains, suggesting that RVFV has fewer functional constraints at this region of the virus envelope. By dissecting the molecular determinants of mAb recognition of Gn, this integrated analysis refines strategies needed for the rational design of vaccines that can elicit a potent and species-wide protective antibody immune response to this important re-emerging pathogen.

## Author summary

Rift Valley fever virus (RVFV) constitutes a continued threat to human health and animal husbandry. While the humoral response against RVFV in humans is known to elicit potently neutralizing and protective antibodies, a comprehensive understanding of the molecular determinants of neutralization remains incompletely defined and is essential for addressing this threat. Here, we describe a protective human monoclonal antibody (mAb) termed RVFV-379, which is encoded by a kappa light chain gene and potently neutralizes RVFV. We structurally characterize this unique mAb in complex with a major antigenic target on RVFV, the Gn glycoprotein, and reveal a binding pose distinct to other reported human mAbs directed against Gn. Through examination of the specificity underlying this antibody-antigen interaction, we refine our knowledge about a key site of vulnerability on the RVFV surface. Further, this integrated functional and structural study provides a template that informs upon determinants of cross-protection against prevalent RVFV strains, an essential consideration for rational vaccine design.

## Introduction

Rift Valley fever virus (RVFV) is an arthropod-borne phlebovirus (family *Phenuiviridae*, order *Bunyavirales*) that mainly infects mosquitoes and ruminants. The virus was first identified in sub-Saharan Africa, but now it has spread into other regions of Africa, some of the southwest Indian Ocean islands, and the Arabian Peninsula. The geographical range of RVFV is expected to extend further following the climate-change-driven spread of its arthropod vectors [1]. These vectors mainly consist of a variety of mosquitoes but also include ticks and hematophagous flies [2]. RVFV outbreaks cause high fetal and neonatal mortality in domestic ruminants, negatively impacting local economies [3,4]. The virus can be transmitted to humans through exposure to contaminated livestock products, such as excrement, blood, undercooked meat, raw milk, and occasionally also via arthropod vectors [5]. Disease symptoms vary from mild fever, weakness, and nausea to more serious conditions, such as meningoencephalitis, hemorrhagic fever, and retinal lesions, which may lead to permanent loss of vision [3,6,7]. No therapeutics or vaccines licensed for use in humans are currently available.

RVFV virions contain a tripartite, single-strand RNA genome consisting of an ambi-sense S segment and negative-sense M and L segments. Two M-encoded type-I transmembrane glycoproteins, RVFV Gn and RVFV Gc, extend from a lipid bilayer membrane and are key targets of the antibody-mediated immune response to infection and immunization [8–12]. RVFV Gn is composed of an N-terminal signal peptide, Gn stem region, and C-terminal head region (Gn^H^) [10,13]. The Gn^H^ structure comprises three domains termed A, B, and β-ribbon [10]. The same fold and domain arrangement is also found in the Gn proteins of the more closely related hantaviruses (order *Bunyavirales*) [14,15] and the envelope protein 2 (E2) of alphaviruses [16]. Pre- and post-fusion structures of the RVFV Gc ectodomain region revealed that the protein forms a class II fusion fold composed of three domains (termed I − III) [17,18]. This fusogen architecture also has been observed in other bunyaviruses, including orthobunyaviruses [19], hantaviruses [20–22], and other phenuiviruses [23]. RVFV Gn and Gc form heterodimers on the envelope surface, which assemble into ring-like pentamers and hexamers in a T = 12 icosahedral lattice [10,24,25]. Fitting RVFV Gn^H^ and Gc crystal structures into cryo-electron microscopy (cryoEM) derived reconstructions of RVFV virions indicates that RVFV Gn^H^ is membrane distal and shields hydrophobic fusion loops on domain II of RVFV Gc [10].

Several cellular receptors have been shown to facilitate RVFV host-cell entry, including the C-type lectin receptor dendritic cell-specific intercellular adhesion molecule-3-grabbing non-integrin (DC-SIGN) [26], heparan sulphate [27], and low-density lipoprotein receptor-related protein 1 (LRP1) [28]. Following endocytosis, RVFV Gc mediates fusion of the viral envelope with intracellular, endosomal membranes [10], enabling delivery of the viral genome and polymerase into the cytosol, allowing transcription and replication [29].

Although both anti-Gn and anti-Gc antibodies are found in naturally infected and immunized individuals, anti-Gn antibodies generally display higher neutralizing potency [11,30,31]. Within Gn^H^, domain A appears to be the dominant target of antibodies in natural infections [30,31], although antibodies also can be induced by domain B (using RVFV Gn^H^ as an immunogen) [32]. The structural basis for mAb-mediated neutralization of RVFV through targeting of RVFV Gn has been reported for several human and animal-derived mAb [30,32,33].

RVFV-268 was identified in a discovery program that generated a set of 20 mAbs derived from B cells of immune individuals [31,33]. This mAb was isolated from an individual immunized with the RVFV MP-12 investigational live attenuated vaccine strain and exhibits exceptionally potent neutralizing activity. RVFV-268 appears to function by bivalently engaging adjacent domain A-localized epitopes on the lattice [33]. RVFV-379, another antibody from the same study, originated from a naturally infected individual. RVFV-379 exhibits potent neutralizing activity [31]. Notably, RVFV-379 is the only reported human antibody with a kappa light chain that targets the RVFV Gn surface protein. MAb RVFV-268 more potently neutralizes RVFV than mAb RVFV-379, with IC_50_ values against the live attenuated vaccine strain MP-12 of ~0.1 ng/mL or 1.3 ng/mL, respectively [31]. Additionally, the IC_50_ values against the wild-type virus strain ZH501 were previously reported as ~0.2 ng/mL or 4.6 ng/mL, for RVFV-268 or RVFV-379 respectively [31]. Previously performed affinity measurements have demonstrated similar binding kinetics for these two mAbs to a recombinant Gn^H^ protein [33]. Competition-binding and epitope mapping studies have demonstrated both antibodies recognize a common major antigenic site [31,33]. In this study, we define the molecular and structural determinants of recognition of RVFV by mAb RVFV-379 and compared the features of this molecular recognition to those of other potently neutralizing human mAbs targeting Gn.

Increasingly, high-resolution structures of neutralizing antibodies in complex with viral antigens inform our understanding of the molecular and structural basis for virus neutralization. In particular, the angle by which an antibody binds to its cognate epitope may impact the degree to which the antibody can neutralize its target. Examples of angular determinants of functional activity include antibodies against HIV-1 [34], SARS-CoV-2 [35,36], RSV [37], Marburg virus [38,39], and Ebola virus [40]. Approach angle can modulate the activity of antibodies that work by diverse mechanisms, including fusion inhibition and direct or indirect receptor blocking. Despite knowledge from study of other viruses, the structural determinants by which human antibodies recognize bunyaviruses such as RVFV remain poorly understood. This gap in knowledge stems in part from the complexity of the Gc-Gn heterodimer structure and the difficulty in obtaining high-resolution structures of these complex antigens. Here, we aimed to define the structural basis of RVFV Gn^H^ recognition and approach angle by the genetically unique human mAb RVFV-379.

Here, we report the crystal structure of RVFV-379 Fab in complex with RVFV Gn^H^. We further define the molecular footprints of potently neutralizing human antibodies to the Gn head domain. The antibody mediated robust protection at low doses from wild-type RVFV challenge when used as therapy in a stringent mouse model of infection. These data offer support for the potential use of this antibody as a prophylactic or therapeutic countermeasure while providing meaningful insights into the structural basis for potent RVFV neutralization by human antibodies.

## Results

### Structure of RVFV-379 bound Gn^H^

The human mAb RVFV-379 was derived from the B cells of an individual in Kenya with prior history of infection with RVFV. The mAb is potently neutralizing (IC_50_ value of 4.6 ng/mL or 1.3 ng/mL against the ZH501 wild-type or investigational vaccine strain RVFV MP-12, respectively) [31]. The RVFV-379 heavy chain (HC) is encoded by IGHV4–61*02 and IGHJ5*02 gene segments (with 91.75% V-region nucleotide homology to V-gene) and the kappa light chain (LC) is encoded by IGKV1–39*01 and IGKJ5*01 gene segments (92.83% V-region nucleotide homology to V-gene) (S1 Fig).

**Fig 1:**
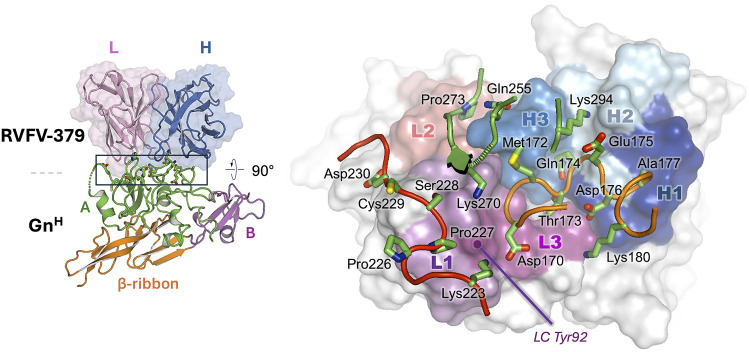
Structure of the antibody RVFV-379 complex with the Gn^H^ ectodomain. **Left panel:** Gn^H^ is shown in cartoon format, with the A, B, and ribbon domains in green, violet, and orange, respectively. The variable regions of RVFV-379 are in transparent-surface and cartoon representation and are highlighted by the black box. **Right panel:** Detailed view of the RVFV-379-Gn^H^ interface. The Fab is shown in surface representation. The CDR-L regions are colored purple (L1), orange (L2), and magenta (L3), and the CDR-H regions dark blue (H1), pale blue (H2), and marine blue (H3). Fab-interacting residues on the Gn^H^ are shown in cartoon representation. The sidechains of relevant residues are displayed as sticks. The majority of Gn^H^ residues involved in Fab binding belong to two loops. Residues in loop Lys223 − Asp230 (red) interacts with the light chain of RVFV-379. Residues in loop Asp170 − Lys180 (orange) predominantly interacts with the heavy chain, but also with some CDR-L3 residues. In addition to residues on these loops, Lys270 and Pro273 interact with the light chain, and Gln255 and Lys294 with the heavy chain. The purple dot denotes the position of the hydroxyphenyl ring of RVFV-379 LC residue Tyr92.

Recombinantly-derived RVFV Gn^H^ protein based on the sequence of the KEN07-KLF112 strain (which is identical to the wild-type strain ZH501 Gn^H^ sequence in the expressed region) was complexed with the Fab fragment of RVFV-379, and a 2.1 Å structure was obtained by X-ray crystallography (Fig 1 and S1 Table). Two near-identical complexes were observed in the asymmetric unit of the crystal with a root mean square deviation (RMSD) value of ~0.35 Å (over 277 Cα atoms) for the Gn^H^ chains. RVFV Gn forms the expected three domain globular fold architecture that resembles the Gn from hantaviruses [22] and the E2 of alphaviruses [16], and consists of domain A, domain B, and a β-ribbon domain (Fig 1, left panel). The most pronounced difference between the two RVFV Gn^H^ structures in the asymmetric unit localizes to the long loop between residues 377–394 of domain B. In one of the Gn^H^ molecules (chain A in the PDB file), as in previously reported Gn^H^ structures, this loop cannot be built due to poor density. However, because of stabilizing crystal contacts, the loop is visible in the other molecule (chain B), revealing a small helix (residues 388 − 393) (S2 Fig).

**Fig 2:**
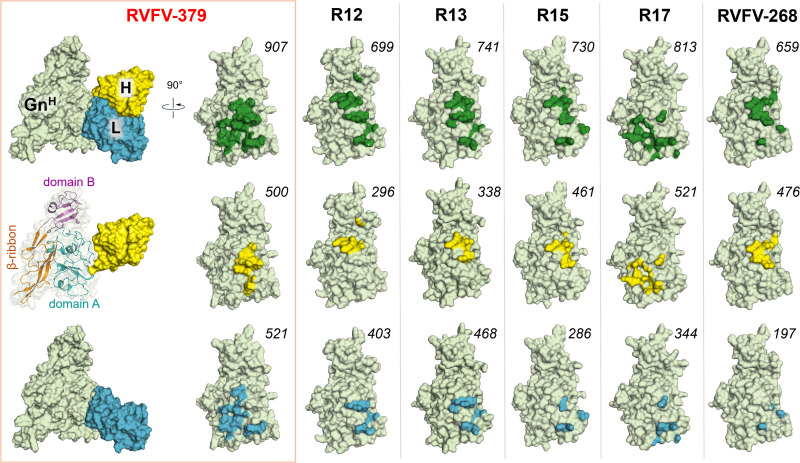
Mapping mAb footprints on RVFV Gn^H^. Surface representation of RVFV Gn^H^ ectodomains (pale green) from all structurally determined Fab complexes with domain **A.** From left to right, complexes with RVFV-379 (379, PDB code 9I59), R12 (6IEK), R13 (6IEA), R15 (6IEB), R17 (6IEC), and RVFV-268 (8AWM). The Fabs are only shown for the RVFV-379 Gn^H^ complex (right panel, with the heavy chain in yellow and the light chain in blue). The footprints (interactions within 4.0 Å) of the combined heavy and light chains on Gn^H^ are shown in dark green (top row). Those of the heavy chains by themselves are in yellow. Footprints of the light chains (bottom row) are in blue. The numbers in italic denote the buried surface area (Å^2^) for each complex, calculated using Chimera X.

As predicted by previous site-directed mutagenesis and competition-binding assays [31], RVFV-379 interacts with domain A of RVFV Gn. The antibody footprint of RVFV-379 (~910 Å^2^) is more extensive than that of other structurally characterized domain-A binding antibodies (average of ~730 Å^2^, Fig 2). Although paratope residues in the heavy chain of RVFV-379 form a substantial interaction with RVFV Gn (~500 Å^2^), the contribution of the cognate light chain residues is also extensive and encompasses a larger area (520 Å^2^) than the corresponding light chains of other characterized mAbs (average of 340 Å^2^) (Figs 1 and 2). Central to the interface is Tyr92 within the complementary determinant region (CDR) 3 of the RVFV-379 LC (CDR-L3). Tyr92 penetrates RVFV Gn^H^ furthest and forms a hydrogen bond with Lys223 of domain A. The interface is further stabilized by 10 additional hydrogen bonds and 3 salt bridges (S3 Fig).

**Fig 3:**
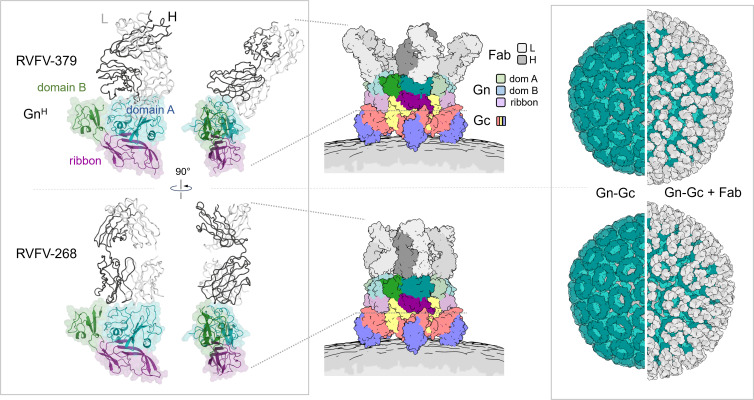
Comparison of the binding of antibodies RVFV-379 and RVFV-268 to Gn^H^. **Left panel:** The structure of the N-terminal ectodomain region of RVFV Gn^H^ (cartoon plus transparent-surface representation) in complex with the Fab of RVFV-379 (ribbon) is shown in two different orientations (top half of the figure) and is compared to that in complex with RVFV-268 (bottom half), showing a ~ 40° angle between the longitudinal axes of the 2 Fabs. **Middle panel:** Fabs of RVFV-379 (top) and RVFV-268 (bottom) modelled onto the higher-order pentameric RVFV Gn − Gc assembly (PDB code 6F9F). Protein chains are colored as defined in the key. **Right panel:** A model of the Fabs (white and grey surface representation for the L- or H-chains, respectively) bound to the icosahedral RVFV Gn − Gc assembly (surface representation, PDB code 6F9B) with Gn in teal, Gc in light teal and the virion membrane in grey. Modeling was performed by superposing the position of the Fab from RVFV-379 and RVFV-268 onto the binding position of each RVFV Gn. In this analysis, no clashes were observed, revealing that full Fab occupancy is allowable.

When compared to previously structurally characterized mAbs, five of the reported structures present epitopes that overlap with that of RVFV-379. Further each of these mAbs exhibits a neutralization potency indicating that this is a commonly targeted neutralization-sensitive site on the surface of RVFV Gn. Interestingly, however, RVFV-379, distinguishes itself from the highly potent and protective mAb RVFV-268, which binds the Gn in a radial orientation. Although, RVFV-379 approaches at an acute angle relative to the membrane (Fig 3) similar to that utilized by the human mAb R17, their heavy and light chain orientation differs [30] (S4 Fig).

### Neutralization sensitivity of potent antibodies to polymorphisms in Gn of diverse strains of RVFV

The antigenic diversity observed across the RVFV surface glycoproteins of known isolates poses a challenge to the development of broad-spectrum mAb-based therapies. RVFV Gn exhibits a higher level of amino acid sequence variation in field strains than the cognate Gc [32]. Despite known variations in the sequence of Gn glycoprotein, the impact on recognition by human antibodies in serum responses or human mAbs is not known. Additionally, RVFV-140 and RVFV-268 were induced by vaccination with RVFV MP-12 investigational live attenuated vaccine strain, while RVFV-379 was induced by infection with an unknown wild-type field strain. To understand how Gn amino acid variations impacts neutralization, we focused on two potently neutralizing antibodies, RVFV-379 and RVFV-268, for which we could integrate structural studies and neutralization data against diverse field strains. Using the crystal structure reported here and the previously described structure of RVFV-268 in complex with Gn^H^ [33], we identified residues with natural variation in close proximity (within 5 Å) to the paratope-epitope interaction (S2 and S3 Tables). We then identified and tested RVFV-268 and RVFV-379 against two available RVFV strains (RVFV Smithburn and Kenya_56_IB8), which exhibit amino acid variation at positions for residues directly interacting with RVFV-379 (S5 Fig) or RVFV-268.

**Fig 4:**
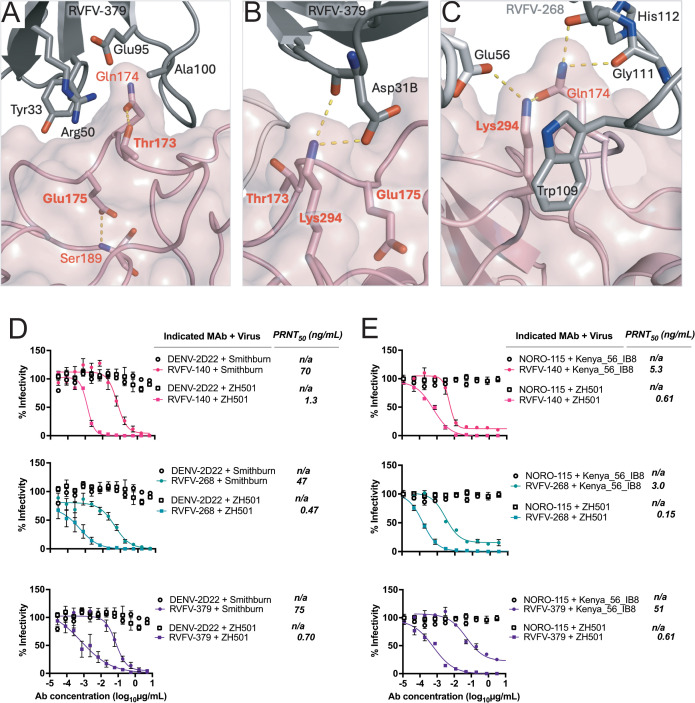
Effect of Gn^H^ polymorphisms in RVFV field strains on mAb RVFV-379 recognition and neutralization. **A.** The interaction between residues Glu175 and Thr172 (bold labels) of Gn^H^ domain A (mauve) and the heavy chain of RVFV-379 (grey). Sidechains of selected residues are shown as sticks with mainchains shown when relevant. The Glu175 sidechain turns inwards and creates a pocket on the surface of domain A that accommodates Tyr33 of the RVFV-379 heavy chain. A E175G mutation, observed in the Kenya_56_IB8 strain, likely changes the trajectory and destabilizes the loop at position 175, leading to a weaker interaction with RVFV-379 [33]. Similarly, T173L, which is present in KEN/Bar-035/07, KEN/Bar-032/07, and KEN/Mal-032/07, is likely to indirectly weaken RVFV-379 binding as Thr173 forms a hydrogen bond that stabilizes the Gln174 sidechain in a position needed to interact with several H-chain residues. **B.** Lys294 is spatially close to both Thr173 and Glu175 (as presented in panel *A*) and forms two hydrogen bonds with Asp31B of the H chain. **C.** Interactions between residues Lys294 and Gln174 of Gn^H^ domain A with RVFV-268. The Lys294 sidechain forms a hydrogen bond with L-chain residue Glu56 and hydrophobic contacts with H-chain residue Trp109. Gln174 interacts with the mainchain of L-chain residues Gly111 and His112. **D.** Neutralization sensitivity of antibodies RVFV-268, RVFV-379, and RVFV-140 against the ZH501 or Smithburn strains of RVFV. The Smithburn strain contains the double mutation D230N/K294E. IC_50_ values were calculated using a sigmoidal-4PL nonlinear fit. Curves reflect data from triplicate wells in two independent experiments. Data are presented as mean values + / − SEM. **E.** Neutralization sensitivity of antibodies RVFV-268, RVFV-379, and RVFV-140 against the ZH501 or Kenya_56_IB8 strains of RVFV. The Kenya_56_IB8 strain contains the mutation Glu175Gly. IC_50_ values were calculated using a sigmoidal-4PL nonlinear fit. Curves reflect data from triplicate wells except RVFV-379 against Kenya_56_IB8 which was performed in duplicate. Data are presented as mean values + / − SEM.

An E175G variation on RVFV Gn domain A, as observed in the Kenya-56_IB8 strain, was previously shown to decrease antibody binding of mAbs RVFV-379 and RVFV-268 using monomeric Gn recombinant protein [33]. While the sidechain of Glu175 does not form direct contacts with RVFV-379, it is instrumental in shaping a pocket that accommodates Tyr33 of the RVFV-379 HC (Fig 4A and S3 Table). Replacing Glu175 with a Gly likely affects the shape or existence of the pocket, locally abolishing antibody binding. Additionally, double mutation of D230N and K294E, which is observed singularly in the Smithburn strain of RVFV, also impacts the binding of several mAbs using monomeric Gn^H^ and mAb in bio-layer interferometry binding studies [33]. In the case of mAbs RVFV-379 and RVFV-268, the D230N mutation is likely to have minimal effect upon binding. Indeed, Asp230 of the Gn forms distant, yet minor contacts with LC paratope residue Phe67 of RVFV-379, and this interaction is unlikely to be affected by the Asp-to-Asn mutation. Conversely, Lys294 forms two hydrogen bonds with Asp31B of the RVFV-379 HC, and the substitution of Lys294 with the negatively-charged Glu likely abolishes this interaction (Fig 4B and S3 Table). Similarly, the K294E mutation likely impacts RVFV-268 binding, to which Lys294 normally contributes via a hydrogen bond with LC residue Glu56 and through hydrophobic interactions with the aliphatic sidechain components of HC residue Trp109. Finally, a hydrogen bond between Lys294 and Gln174 may stabilize both these domain A residues in their positions in the unmutated Gn^H^ (Fig 4C and S3 Table). Consistent with these structure-based observations, neutralization of RVFV by mAbs RVFV-268, RVFV-379, and RVFV-140 was greatly impacted by the presence of the D230N and K294E double mutation in the Smithburn strain. Surprisingly, despite previously reported complete loss of binding by RVFV-268 to recombinant Gn^H^ containing K294E from the Smithburn strain, the antibody retained significant neutralization (100-fold increase in IC_50_ value) against authentic Smithburn strain in a live virus assay as compared to the expected outcome of abolished neutralization (Fig 4D). Similarly, these antibodies were assessed for their neutralization capacity against the RVFV strain, Kenya_56_IB8, which contains the E175G variation. As expected, we observed a significant reduction of RVFV-379 neutralization potency compared to that obtained with RVFV strain ZH501 (a change of IC_50_ value from < 2 to 51 ng/mL) (Fig 4E). RVFV-140 was included in these experiments as a control mAb that recognizes a different antigenic site in the case that these strains abolished neutralization against the test mAbs. The epitope for the potently neutralizing human mAb RVFV-140 has not been determined yet, as it does not bind to recombinant Gc or Gn proteins, but most likely recognizes a complex quaternary antigen on the virion surface.

### Robust therapeutic protection against wild-type RVFV challenge

**Fig 5:**
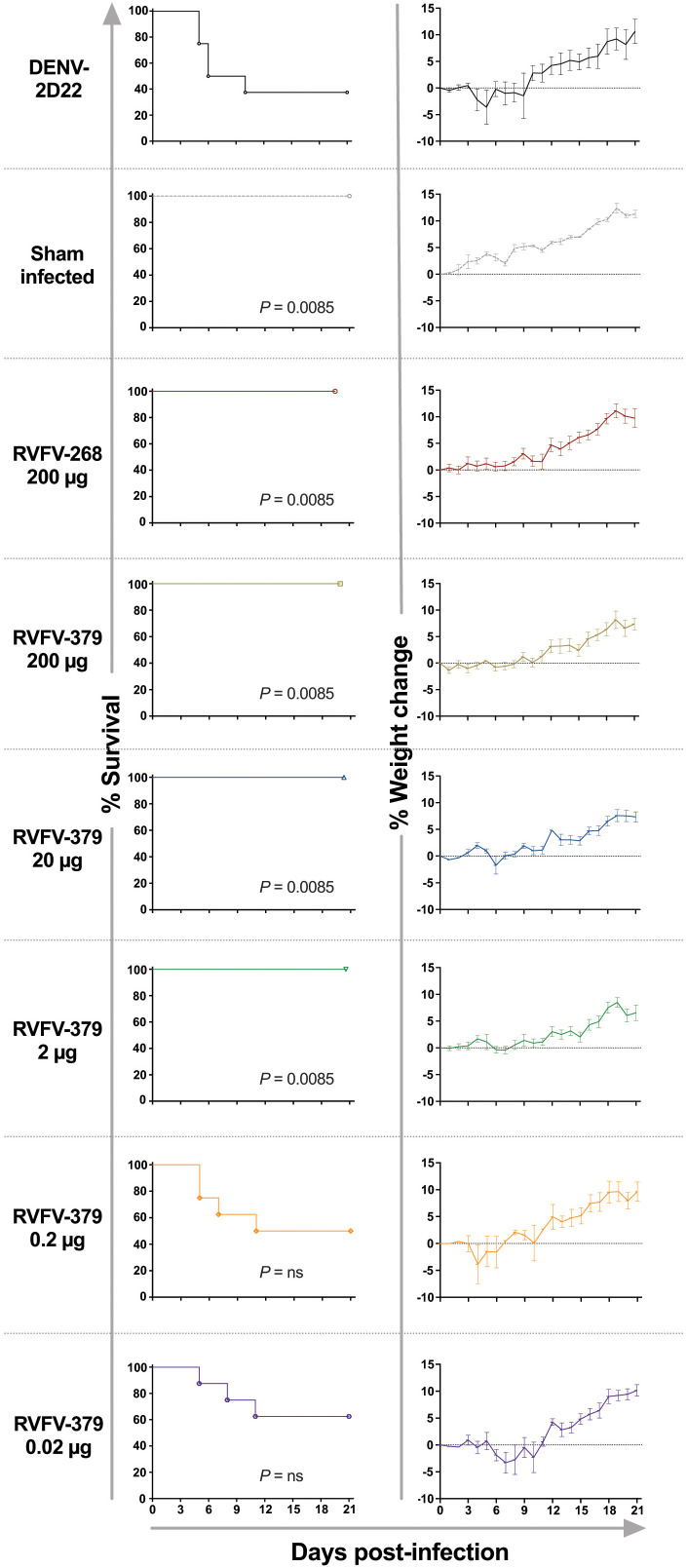
Mab RVFV-379 is effective therapeutically at low doses against RVFV in a stringent *in vivo* model of infection. MAbs were administered once by the IP route to mice (n = 8 per group) at 2 d.p.i. Infection occurred following subcutaneous inoculation of 100 PFU of RVFV strain ZH501. MAbs RVFV-379, RVFV-268, or controls were tested. Doses administered are denoted in the figure in a 10-fold sequential dose down. Kaplan-Meier survival curves were statistically analyzed using a log-rank (Mantel-Cox) test where treated animals (**P < 0.01, *P < 0.05) were compared to animals treated with DENV-2D22 negative control. Weight graphs reflect group means and standard error of the means of the percent change in weight of animals relative to the weight on the day of the virus challenge. Sham-infected no-virus controls are shown. P values for each condition tested compared to the DENV-2D22 control treated group using a Log-rank (Mantel-Cox) test: no-virus control – 0.0085; 200 µg RVFV-268 – 0.0085; 200 µg RVFV-379– 0.0085; 20 µg RVFV-379– 0.0085; 2 µg RVFV-379– 0.0085; 0.2 µg RVFV-379 – not significant; 0.02 µg RVFV-379 – not significant. Median day to death (MDD) are as follows: no-virus control – all animals survived to end of study; 200 µg RVFV-268–all animals survived to end of study; 200 µg RVFV-379– all animals survived to end of study; 20 µg RVFV-379– all animals survived to end of study; 2 µg RVFV-379– all animals survived to end of study; 0.2 µg RVFV-379 – 7; 0.02 µg RVFV-379 – 8.3; 200 µg DENV-2D22 – 6.4. MDD was analyzed by ANOVA with Dunnett’s posttest. Source data are provided as a S1 Data.

To test the capacity of RVFV-379 IgG1 to protect *in vivo*, we tested the mAb in a mouse model of infection using a therapeutic study design. It is interesting to note that the neutralization potency of RVFV-379 is nearly 10-fold weaker than that of RVFV-268. RVFV-268 therapeutic efficacy has been extensively characterized previously in this animal model [31,33], which revealed RVFV-268 can mediate a therapeutic effect at ultra-low doses (0.2 µg/mouse). Here, we compared the protective capacity of RVFV-379 with the more potently neutralizing RVFV-268. We observed robust protection against RVFV wild-type strain ZH501 infection (note, this strain does not contain the Smithburn and Kenya_56_IB8 strain-specific mutations tested in the previous section) at low doses (2 µg per mouse) of RVFV-379 IgG1 relative to the control-mAb-treated animals (Fig 5). This result is about 10-fold inferior to that of RVFV-268, consistent with the differences in IC_50_ values. RVFV-379 IgG1 provided protection against weight loss (Fig 5) and reduced viremia in the serum, liver, and spleen of challenged animals after treatment with low doses of antibody (S6 Fig). At a 0.2 µg dose per mouse, viral titers were reduced in the liver and spleen but not the serum, and protection from death and weight loss was lost (Figs 5 and S6).

## Discussion

In this work, we defined the genetic, structural, and functional features that drive recognition of RVFV Gn^H^ by RVFV-379. Comparing two antibodies that have similar affinities for their epitope and similar epitopes (as defined by previous low-resolution analyses [31, 33]), and that compete with one another for their cognate epitope, presented a unique opportunity to further define the molecular recognition patterns for potently neutralizing antibodies on the RVFV Gn^H^ domain A.

We performed detailed *in vitro* experiments using divergent strains of RVFV to provide quantification of the extent to which these different antibodies are sensitive to naturally occurring mutations in the epitope. Previous studies have shown that the binding affinity of RVFV-379 for recombinant RVFV Gn decreases following E175G (a naturally occurring variation in the Kenya_56_IB8 strain) or T173L mutations [33]. Our X-ray crystal structures suggest that these mutations alone would not affect RVFV-379 recognition unless they introduce changes to the local structure of RVFV Gn. This occurrence is plausible, especially in the case of Glu175, which presents a buried side chain that does not interact with the Fab but instead helps to form a pocket that accommodates Tyr33 of the RVFV-379 HC (Fig 4A). Indeed, a E175G substitution may alter the trajectory of the loop on which it resides, destroying the pocket and causing steric hindrance for Tyr33, which in turn could cause mismatches elsewhere in the complex. Reduced binding also was reported with a double mutant K294E and D230N variant [33]. Asp230 is distal from the mAb-Gn interface and thus the mutation does not have a direct effect upon the interaction, however Lys294 occupies an acidic pocket on the Gn^H^ surface. Changing the lysine to a negatively-charged glutamic acid would likely introduce repulsion to RVFV-379 at this site (Fig 4B).

The K294E mutation has a much greater effect upon RVFV-268 binding compared to RVFV-379 [33]. The larger footprint of RVFV-379 may, in part, be responsible for this finding, as a greater number of additional interactions may mitigate the effect of the Lys294 mutation. Lys294 may play a more important role in RVFV-268 binding compared to that of RVFV-379, since RVFV-268 forms a hydrogen bond with Glu56 of the LC and a hydrophobic interaction with Trp109 of the HC. Lys294 also interacts with the nearby Gn^H^-residue Gln174 that is also involved in RVFV-268 binding. The resulting, mutual stabilization of the Lys294 and Gln174 sidegroups may strengthen their interaction with the Fab. Despite the K294E mutation in Smithburn abolishing binding to monomeric Gn, the fact that Smithburn strain of RVFV is still neutralized albeit at 100-fold higher concentration of mAb, is promising for the effectiveness of RVFV-268 as a therapeutic. The mechanism by which RVFV-268 retains neutralization of Smithburn is not fully known, although we speculate that the bivalent nature of an IgG molecule may have a role to play. In the complex with RVFV-379, Lys294 only interacts with HC residue Asp31B, albeit using two hydrogen bonds.

Notably, antibodies against RVFV targeting the head domain of Gn, to which potently neutralizing antibodies bind, typically are encoded by a lambda light chain. To date, RVFV-379 is the only known mAb encoded by a kappa light chain that recognizes this site of vulnerability. Here, we report the first structure to define the genetic and structural features of a unique human mAb against RVFV that uses a kappa light chain.

In summary, human antibodies to RVFV Gn can mediate very potent neutralizing effects that correspond to their level of efficacy *in vivo*. Through structural comparison of RVFV Gn in complex with different human mAbs, it is tempting to suggest that the angle of binding of human mAbs to the dominant antigenic site of neutralization vulnerability on RVFV Gn^H^ is an important determinant of neutralization potency. However, given that mAb R15 binds at a radial orientation similar to RVFV-379 yet exhibits a low neutralization potency, it is important to consider the contribution of other variables, such as epitope specificity, steric hinderance to epitope accessibility introduced by the flexible mAb constant region, and the potential for mAb-induced disruption the Gn-Gc capsomeric assembly. Indeed, additional studies that dissect to what extent unique approach angles impart an effect on neutralization potency are warranted. Additionally, the isolation and characterization of more human mAbs to Gn^H^ could further define the structural determinants of potent neutralization of RVFV. Future efforts to characterize the capacity of these and other antibodies that bind in different angles to bivalently interact with Gn on the viral surface will further elucidate the mechanism of neutralization. These results could aid in the optimization of rational vaccine designs for RVFV by providing a blueprint for the optimal presentation of protective epitopes that induce potently neutralizing antibodies. Additionally, polyclonal antibody neutralization magnitude against RVFV should be appreciated in the context of complex antigen recognition angles within Gn^H^ domain A.

## Materials and methods

### Ethics statement

Work with pathogenic RVFV was approved by the Utah State University Institutional Biosafety Committee and conducted in AAALAC-accredited and Select Agent-approved animal (A)BSL-3 + laboratories. All animal procedures complied with USDA guidelines and were conducted under protocol #10248, approved by the Utah State University Institutional Animal Care and Use Committee. RVFV ZH501 animal studies were conducted at Utah State University in biosafety level-3 enhanced (BSL-3+) approved facilities using appropriate powered air-purifying respirators and personal protective equipment. RVFV wild-type strain ZH501 was kindly provided by Dr. Stuart Nichol at the CDC in Atlanta, Georgia, USA.

### Cell lines

Vero E6 (ATCC: CRL-1586) were maintained at 37°C in 5% CO_2_ in Dulbecco’s Modified Eagle Medium (DMEM; Thermo Fisher Scientific) containing 10% (v/v) heat-inactivated FBS (HyClone).

### Viral lots

RVFV ZH501 animal studies were conducted at Utah State University in biosafety level-3 enhanced (BSL-3+) approved facilities using appropriate powered air-purifying respirators and personal protective equipment. RVFV wild-type strain ZH501 was kindly provided by Dr. Stuart Nichol at the CDC in Atlanta, Georgia, USA. The RVFV Smithburn strain (obtained from Christina Spiropoulou at CDC, Atlanta) and ZH501 (obtained from Stuart Nichol, CDC, Atlanta) neutralization strain testing was performed at the University of Pittsburgh Regional Biocontainment Laboratory within a Select Agent-approved BSL-3 facility. The RVFV_IB8 neutralization strain testing was performed at UTMB in BSL-3 facilities. The viral stock was obtained from the Viral Special Pathogens Branch at the Centers for Disease Control and Prevention (Atlanta, GA).

### Animal models

Seven- to eight-week-old male and female BALB/c mice were obtained from Charles River Laboratories (Wilmington, MA). Mice were housed in microisolator cages and provided water and food *ad libitum*. The mouse RVFV challenge efficacy studies were approved by the Utah State University Institutional Biosafety Committee and conducted in Select Agent-approved animal (A)BSL-3 + facilities.

### Antibody production and purification

For recombinant mAb production, the heavy and light chain antibody variable genes were synthesized into cDNA and cloned into a full-length IgG1 DNA plasmid expression vector [41]. The vector was transformed into *Escherichia coli* cells to produce large amounts of DNA. Following the manufacturer’s protocol, plasmids encoding heavy and light antibody chains were transiently transfected into Expi293F cells to produce mAb proteins. Secreted IgGs were purified from filtered supernatants by affinity chromatography using protein G columns (GE Life Sciences, Protein G HP columns) on an ÄKTA pure instrument. Purified mAbs were processed by buffer-exchanging into PBS, filtered using sterile 0.45 μm Millipore filter devices, concentrated, and stored at −80°C.

### Fab production and purification

DNAs encoding the heavy and light chain variable regions of antibody fragments were inserted into DNA plasmid expression vectors encoding Fabs, processed, and transiently expressed similarly as full-length IgG expression. Fab fragments were purified using CaptureSelect CH1-XL columns (Thermo Fisher Scientific) on an ÄKTA pure instrument. Final Fab fragments were buffer-exchanged to PBS, concentrated, and frozen in an ethanol-dry ice bath and shipped to Oxford University from Vanderbilt University.

### RVFV Gn^H^ protein expression and purification

A his-tagged RVFV Gn^H^ construct (GenBank ID ABR28386.1; AA154–468) was expressed as a secretory protein in HEK293T cells as described before [13]. The expression medium (Dulbecco’s Modified Eagle Medium; GIBCO) was clarified (4,000 × *g* for 20 min at 15°C), supplemented with extra NaCl (to 600 mM), imidazole (8 mM) and Tris pH 8.0 (10 mM), filtered using a 0.45 μm cut-off vacuum filtration unit (Nalgene) and brought over a 5-mL HisTrap Excel column (Cytiva). The column was washed with 500 mM NaCl in 10 mM Tris pH 8.0, before protein was eluted with the same buffer supplemented with imidazole (pH 8.0, to 700 mM). The eluted protein was concentrated using a 30 kDa-MWCO centrifugal filtration device (Merck; 12°C, 3,500 × *g*) and submitted to size-exclusion chromatography (SEC) over a Superdex 200 Increase 10/300 GL column (Cytiva) using 10 mM Tris pH 8.0, 150 mM NaCl as running buffer. The fraction containing monomeric RVFV Gn^H^ was concentrated (as above) and combined with excess amount of RVFV-379. Following a 3 hr incubation at room temperature, the Gn^H^-Fab complex was purified by SEC (as above) and concentrated to 5.5 mg/mL using a centrifugal filtration device (as above), simultaneously exchanging the buffer to 5 mM Tris pH 8.0, 50 mM NaCl.

### Crystallization, data collection, and structure elucidation

Crystals of RVFV Gn^H^ in complex with antibody RVFV-379 were formed at 20.5°C using the sitting drop vapor diffusion method [42] by mixing 100 nL protein (10 mg mL^-1^) with 100 nL of a precipitant solution containing 5% w/v polyethylene glycol 4000, 50 mM MES pH 6.0, and 5 mM MgSO_4_. Glycerol was added to 25% (v/v) for cryoprotection. The Diamond Light Source beamline I24 (Harwell, UK) was used for diffraction data collection at 100K. Data processing employed the XIA2 program suite [43] and the structure of RVFV Gn^H^ in complex with RVFV-379 was solved with the molecular replacement program PHASER [44], using the coordinates of the RVFV Gn^H^ domain [30,33] and of Fabs [45]. COOT [45] was used for model building, and Phenix [46] and BUSTER [47] were for refinement. Structure validation employed COOT and Molprobity [48]. Refinement statistics are given in S1 Table. Figures were prepared using PyMOL Molecular Graphics System (Version 2.1) [49] and UCSF ChimeraX [50]. The antibody-antigen interface was characterized with PISAebi [51], PLIP [52], and LigPlot [53]. The Abnum numbering program [54] was used to number Fab residues according to the Chothia scheme.

### Neutralization assay with Rift Valley fever virus isolates

The assays assessing the human mAbs against ZH501 and Smithburn strains of RVFV were performed as follows at the University of Pittsburgh. MAb neutralization against the RVFV ZH501 strain in comparison to the Smithburn strain was performed on Vero E6 cell (ATCC CRL-1586) monolayer cultures. Briefly, cells were plated on 12-well plates the day before testing. Antibodies then were diluted in D2 medium (DMEM, 2% fetal bovine serum (FBS), 1% L-glutamine, 1% penicillin-streptomycin) to appropriate concentrations following 3-fold serial dilutions and mixed with either the ZH501 or the Smithburn strains amounting to 100 plaques per well for 1 hr at 37°C. Next, 100 μL of the virus-antibody mixture was added to the cells in triplicate and incubated for 1 hr at 37°C to allow viral absorption. Following this, the mixture was removed, agarose overlay (minimum essential medium, 2% FBS, 1% penicillin/streptomycin, 1.5% (1M) HEPES buffer, and 0.8% SeaKem agarose) was added, and the cells were incubated for 3 days at 37°C. Cells were fixed using formaldehyde and counted using crystal violet stain.

The virus neutralization assay assessing the human mAbs against ZH501 and the Kenya_56_IB8 strains of live authentic RVFV were performed in the BSL-3 facility of the Galveston National Laboratory using Vero E6 cells following the standard procedure. Briefly, Vero E6 cells were cultured in 96-well plates (10^4^ cells/well). Next day, 4-fold serial dilutions of antibodies were made using MEM + 2% FBS, as to get an initial concentration of 100 µg/mL. Diluted antibodies (in 60 µL) were mixed gently with equal volumes of RVFV strains Kenya_56_IB8 or ZH501 containing 200 pfu and incubated for 1 hr at 37°C in 5% CO_2_ atmosphere. The virus-antibody mixtures (100 µL) were added to cell monolayers in triplicates and incubated for 1 hr 37°C in 5% CO_2_ atmosphere. Later, virus-antibody mixtures were discarded, and cell monolayers were overlaid with 0.6% methylcellulose and incubated for 3 days. The overlays were removed, the plates were fixed in 4% paraformaldehyde twice and removed from the BSL-3 containment following an approved protocol. The plates were stained with 1% crystal violet, and virus-induced plaques were counted. The percentage of neutralization and/or NT_50_ of antibody were calculated by dividing the plaques counted at each dilution with plaques of virus-only control. For antibodies, the inhibitory concentration at 50% (IC_50_) values were calculated using GraphPad Prism software (version 10.4.1).

### Animal protection studies from infection with wild-type RVFV

Therapeutic assessment of mAbs was performed in groups of 7- to 8-week-old male and female mixed cohort BALB/c mice [55] (n = 8 per the treatment group (4 per group for day 3 sacrificed for viral titers) and n = 5 for sham-infected (2 sacrificed for viral titer controls). Animals were inoculated with 100 PFU for the comparison of mAbs experiment with RVFV strain ZH501 by the subcutaneous route. Animals were treated with mAb once by intraperitoneal injection on 2 days post infection (d.p.i.). Human mAb DENV 2D22 (specific to an unrelated target, dengue virus) was used as the isotype-matched negative control treatment. Mice were monitored daily from 0 to 21 d.p.i. for survival and body weight. Mice were euthanized 21 d.p.i or when moribund following IACUC-approved protocols.

Viral titers were assayed using an infectious cell culture assay, as previously described [56]. Here, a volume of tissue homogenate or serum was diluted and added to triplicate wells of Vero 76 cell monolayer cultures. Viral cytopathic effect (CPE) was determined 7 days after plating to calculate 50% endpoints. Lower limits of detection (LOD) were 1.49 log_10_ 50% cell culture infectious dose (CCID_50_)/mL in serum and 2.1 log_10_ CCID_50_/g in tissue. In samples for which virus CPE was not detected, the representative value of LOD was assigned for analysis.

### Statistical analysis

Kaplan–Meier survival curves were analyzed using the Mantel-Cox log-rank test. Differences between groups were analyzed by the Fisher’s exact (two-tailed) test. Viremia and mean day-to-death were compared using a one-way ANOVA with Dunnett’s posttest to correct for multiple comparisons test. Weight data are represented as the group mean and standard error of the percent change in weight of surviving animals relative to starting weight. Technical and biological replicates are indicated in the methods and figure legends. Error bars in figures represent standard deviation. Statistical analyses were performed using Prism v9 (GraphPad RRID: SCR_002798).

## Supporting information

S1 Data RVFV 379 paper source data. Source data for Figs 4D, 4E, 5 and S6.
(XLSX)

S1 Fig The antibody variable genes encoding RVFV-379 compared with the inferred germline precursor genes. The antibody variable gene sequences of RVFV-379 were aligned with the inferred germline gene segment sequences. Heavy and light chain sequences are shown separately. FR indicates framework regions; CDR indicates complementarity-determining regions. The residue numbers follow the Chothia scheme of antibody numbering [54] used throughout the paper.
(DOCX)

S2 Fig Superposition of Gn^H^ domain B structures from the asymmetric unit of the crystal. The blue arrow indicates the helical region found in the long loop (His377 to Leu394) in one of the two RVFV-379 Gn^H^ molecules in the asymmetric unit (PDB code 9I59, chain B; blue cartoon). Only one other structure in the database shows the loop (PDB code 5Y0W, orange cartoon), which, however, follows a different trajectory and does not contain a helical region. In the 14 other RVFV Gn^H^ chains deposited to date (in 10 PDB files), the loop was not built, presumably due to poor electron density (as illustrated by 8AWM in the figure).
(DOCX)

S3 Fig The interaction network between mAb RVFV-379 and RVFV Gn. Epitope residues of RVFV Gn are shown above the dashed line with inter-atomic bonds and labels in green. Paratope residues of RVFV-379 Fab are shown beneath the dashed line Labels and inter-atomic bonds are in yellow for loop H2-residues and brown for H3-residues, whilst the corresponding colors for L1-, L2- and L3-loop residues are cornflower blue, sky blue and navy blue, respectively. Analysis was performed using the Ligplot+ (v.2.2.8) program [53,57].
(DOCX)

S4 Fig The angles of approach utilized by human mAbs against RVFV Gn. Cartoon representation showing reported structures of human Fabs in complex with RVFV Gn, highlighting the different angles of approach. RVFV-Gn structures are in green, Fab light chains (L) in blue, and Fab heavy chains in yellow. RVFV mAbs RVFV-379 (PDB ID 9I59 [reported here], IC_50_ ~ 4.6 and ~1.3 ng ml^−1^ [1]), RVFV-268 (8AWM, IC_50_ ~ 0.2 and ~0.1 ng ml^−1^ [31]), R12 (6IEK, IC_50_ = 1.85 ± 1.61 ng ml^−1^ [30]), R13 (6IEA, IC_50_ 56.2 ± 31.5 ng ml^−1^ [30]), R15 (6IEB, IC_50_ = 0.53 ± 0.25 ng ml^−1^ [30]), and R17 (6IEC, IC_50_ = 2.53 ± 2.39 ng ml^−1^ [30]) are shown. The neutralization IC50 values for mAbs R12, R13, R15, and R17 were derived from plaque assays with Vero cells [30]. The IC50 values for RVFV-379 and RVFV-268 were derived from plaque assays with ZH50 *wt* strain and MP-12 vaccine strains, respectively [31].
(DOCX)

S5 Fig Alignment of Gn^H^ proteins of RVFV strains used to test neutralization sensitivity of antibodies. The alignment was generated using ESPript [58]. The Gn^H^ sequence of the ZH-501 strain is identical to that of the KEN07-KLF112 strain used for X-ray crystallography. Secondary structure elements are given above the alignment, the numbers in green denote positions of cysteines forming disulphide bonds. The boxes underneath the alignment denote amino-acids interacting with RVFV-379, and are colored black or grey, for heavy or light chain contacts, respectively.
(DOCX)

S6 Fig Treatment with mAb RVFV-379 reduced viral titers in various organs at low doses. Viral titer data were obtained using an infectious cell culture assay in technical triplicate to assess the efficacy of mAbs in the therapeutic setting. Viral cytopathic effect was used to calculate 50% endpoint values. Lower limits of detection (LOD) were 1.49 log_10_ 50% cell culture infectious dose (CCID_50_)/mL for serum or 2.1 log_10_ CCID_50_/g tissue. In samples presenting with virus below the limit of detection (LOD), the representative value of LOD was assigned for analysis. Human mAb DENV 2D22 (specific to an unrelated target, dengue virus) was used as the isotype-matched negative control mAb. Four animals per group were sacrificed on 3 d.p.i. for analysis of virus in serum (A), liver (B), and spleen (C). The dotted line represents the LOD. Data were analyzed using an ordinary one-way ANOVA correcting for multiple comparisons using a Dunnett’s post-test to compare the differences in viral titer (**P < 0.01, *P < 0.05).
(DOCX)

S1 Table Data collection and refinement statistics. (DOCX)

S2 Table Natural genetic variation and respective frequency of polymorphisms in RVFV Gn^H^ in close proximity of the RVFV-379 paratope of 298 full-length M segments of RVFV. (DOCX)

S3 Table Residues on the surface of Gn^H^ within 5Å of a residue on RVFV-379 heavy (H) or light (L) and their contact type. Empty cells indicate that no contact was observed between RVFV-379 and Gn^H^.
(DOCX)
