# Supplementary material for: Structural basis for recognition of Rift Valley fever virus Gn protein by a human neutralizing monoclonal antibody with a kappa light chain
Source: PLoS Pathog. 2026 Feb 17;22(2):e1013926. doi: 10.1371/journal.ppat.1013926 (PMC12912543; doi:10.1371/journal.ppat.1013926)
Supplement: S1 Fig — The antibody variable gene sequences of RVFV-379 were aligned with the inferred germline gene segment sequences. Heavy and light chain sequences are shown separately. FR indicates framework regions; CDR indicates complementarity-determining regions. The residue numbers follow the Chothia scheme of antibody numbering [54] used throughout the paper. (DOCX) [file ppat.1013926.s002.docx]

**S1 Fig. The antibody variable genes encoding RVFV-379 compared with the inferred germline precursor genes.** The antibody variable gene sequences of RVFV-379 were aligned with the inferred germline gene segment sequences. Heavy and light chain sequences are shown separately. FR indicates framework regions; CDR indicates complementarity-determining regions. The residue numbers follow the Chothia scheme of antibody numbering (54) used throughout the paper.
