# Supplementary material for: Structural basis for recognition of Rift Valley fever virus Gn protein by a human neutralizing monoclonal antibody with a kappa light chain
Source: PLoS Pathog. 2026 Feb 17;22(2):e1013926. doi: 10.1371/journal.ppat.1013926 (PMC12912543; doi:10.1371/journal.ppat.1013926)
Supplement: S2 Fig — The blue arrow indicates the helical region found in the long loop (His377 to Leu394) in one of the two RVFV-379 GnH molecules in the asymmetric unit (PDB code 9I59, chain B; blue cartoon). Only one other structure in the database shows the loop (PDB code 5Y0W, orange cartoon), which, however, follows a different trajectory and does not contain a helical region. In the 14 other RVFV GnH chains deposited to date (in 10 PDB files), the loop was not built, presumably due to poor electron density (as illustrated by 8AWM in the figure). (DOCX) [file ppat.1013926.s003.docx]

**S2 Fig. Superposition of Gn^H^ domain B structures from the asymmetric unit of the crystal.** The blue arrow indicates the helical region found in the long loop (His377 to Leu394) in one of the two RVFV-379 Gn^H^ molecules in the asymmetric unit (PDB code 9I59, chain B; blue cartoon). Only one other structure in the database shows the loop (PDB code 5Y0W, orange cartoon), which, however, follows a different trajectory and does not contain a helical region. In the 14 other RVFV Gn^H^ chains deposited to date (in 10 PDB files), the loop was not built, presumably due to poor electron density (as illustrated by 8AWM in the figure).
