# Supplementary material for: Structural basis for recognition of Rift Valley fever virus Gn protein by a human neutralizing monoclonal antibody with a kappa light chain
Source: PLoS Pathog. 2026 Feb 17;22(2):e1013926. doi: 10.1371/journal.ppat.1013926 (PMC12912543; doi:10.1371/journal.ppat.1013926)
Supplement: S3 Fig — Epitope residues of RVFV Gn are shown above the dashed line with inter-atomic bonds and labels in green. Paratope residues of RVFV-379 Fab are shown beneath the dashed line Labels and inter-atomic bonds are in yellow for loop H2-residues and brown for H3-residues, whilst the corresponding colors for L1-, L2- and L3-loop residues are cornflower blue, sky blue and navy blue, respectively. Analysis was performed using the Ligplot+ (v.2.2.8) program [53,57]. (DOCX) [file ppat.1013926.s004.docx]

**
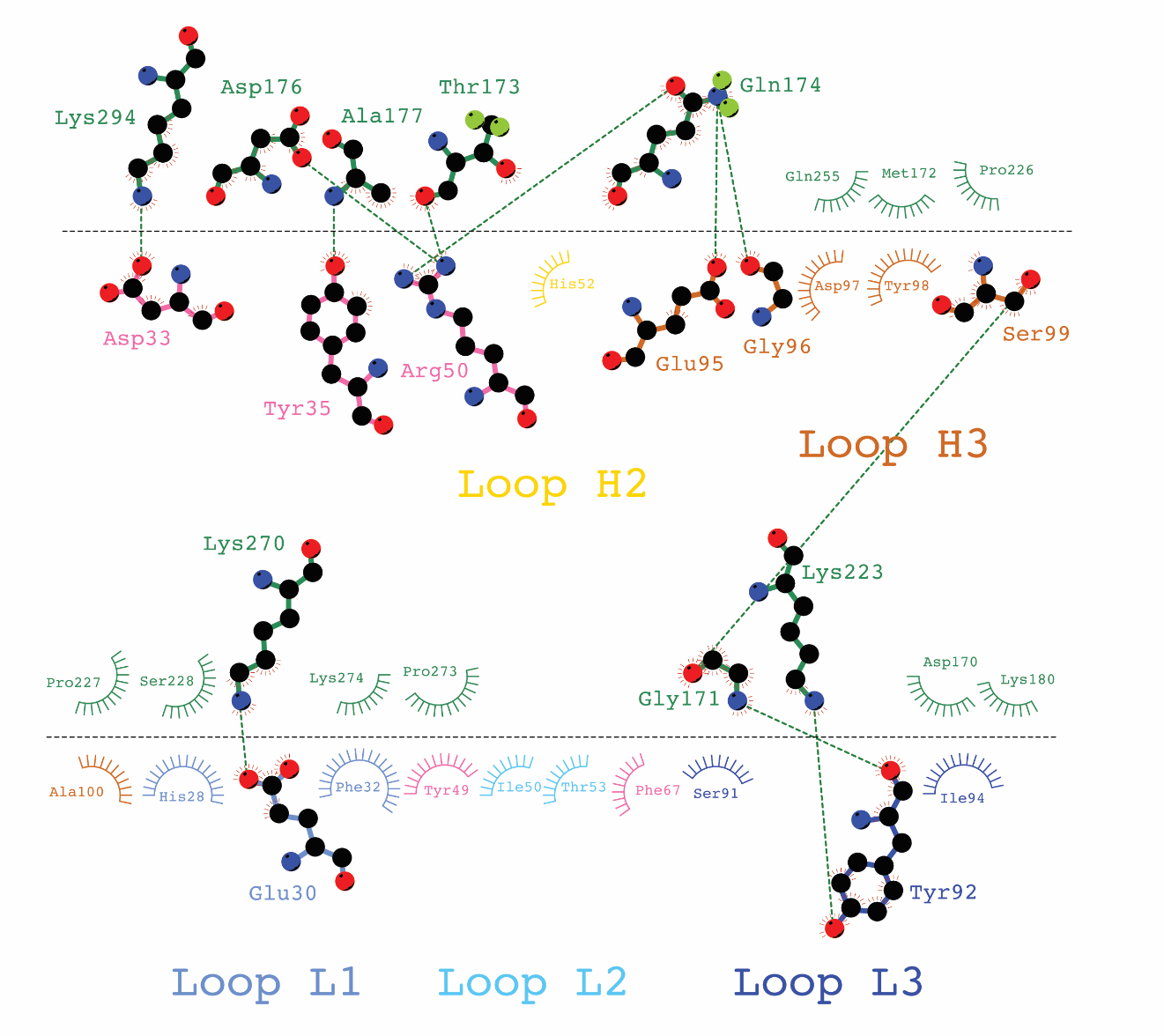
**

**S3 Fig. The interaction network between mAb RVFV-379 and RVFV Gn.** Epitope residues of RVFV Gn are shown above the dashed line with inter-atomic bonds and labels in green. Paratope residues of RVFV-379 Fab are shown beneath the dashed line Labels and inter-atomic bonds are in yellow for loop H2-residues and brown for H3-residues, whilst the corresponding colors for L1-, L2- and L3-loop residues are cornflower blue, sky blue and navy blue, respectively. Analysis was performed using the Ligplot+ (v.2.2.8) program (2, 3).
