# Supplementary material for: Structural basis for recognition of Rift Valley fever virus Gn protein by a human neutralizing monoclonal antibody with a kappa light chain
Source: PLoS Pathog. 2026 Feb 17;22(2):e1013926. doi: 10.1371/journal.ppat.1013926 (PMC12912543; doi:10.1371/journal.ppat.1013926)
Supplement: S4 Fig — Cartoon representation showing reported structures of human Fabs in complex with RVFV Gn, highlighting the different angles of approach. RVFV-Gn structures are in green, Fab light chains (L) in blue, and Fab heavy chains in yellow. RVFV mAbs RVFV-379 (PDB ID 9I59 [reported here], IC50 ~ 4.6 and ~1.3 ng ml−1 [1]), RVFV-268 (8AWM, IC50 ~ 0.2 and ~0.1 ng ml−1 [31]), R12 (6IEK, IC50 = 1.85 ± 1.61 ng ml−1 [30]), R13 (6IEA, IC50 56.2 ± 31.5 ng ml−1 [30]), R15 (6IEB, IC50 = 0.53 ± 0.25 ng ml−1 [30]), and R17 (6IEC, IC50 = 2.53 ± 2.39 ng ml−1 [30]) are shown. The neutralization IC50 values for mAbs R12, R13, R15, and R17 were derived from plaque assays with Vero cells [30]. The IC50 values for RVFV-379 and RVFV-268 were derived from plaque assays with ZH50 wt strain and MP-12 vaccine strains, respectively [31]. (DOCX) [file ppat.1013926.s005.docx]

**
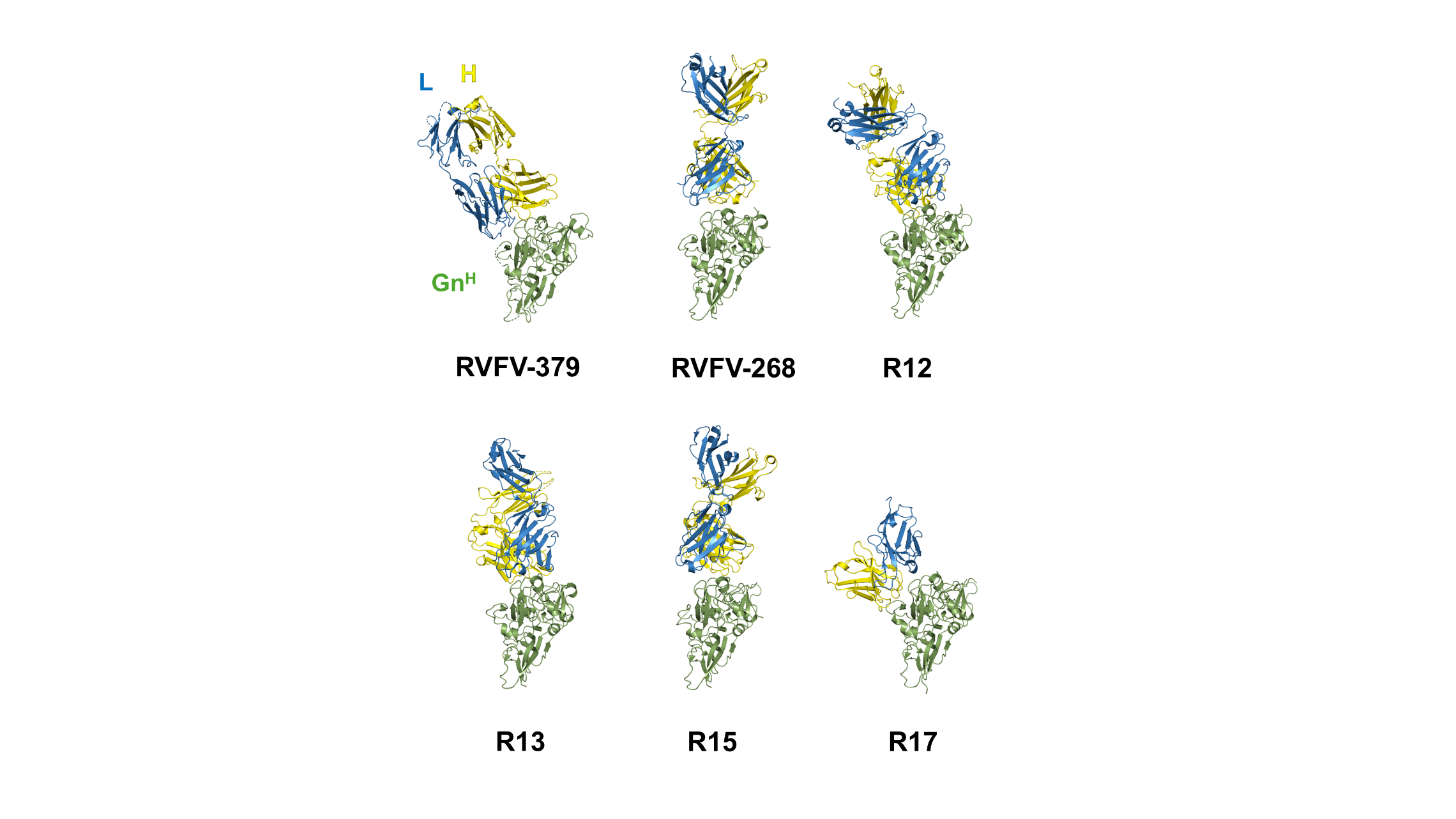
**

**S4 Fig. The angles of approach utilized by human mAbs against RVFV Gn.** Cartoon representation showing reported structures of human Fabs in complex with RVFV Gn, highlighting the different angles of approach. RVFV-Gn structures are in green, Fab light chains (L) in blue, and Fab heavy chains in yellow. RVFV mAbs RVFV-379 (PDB ID 9I59 [reported here], IC_50_ ~4.6 and ~1.3 ng ml^−1^ [1]), RVFV-268 (8AWM, IC_50_ ~0.2 and ~0.1 ng ml^−1^ (4)), R12 (6IEK, IC_50_ = 1.85 ± 1.61 ng ml^−1^ (5)), R13 (6IEA, IC_50_ 56.2 ± 31.5 ng ml^−1^ (5)), R15 (6IEB, IC_50_ = 0.53 ± 0.25 ng ml^−1^ (5)), and R17 (6IEC, IC_50_ = 2.53 ± 2.39 ng ml^−1^ (5)) are shown. The neutralization IC50 values for mAbs R12, R13, R15, and R17 were derived from plaque assays with Vero cells (5). The IC50 values for RVFV-379 and RVFV-268 were derived from plaque assays with ZH50 *wt* strain and MP-12 vaccine strains, respectively (4).
