# Supplementary material for: Structural basis for recognition of Rift Valley fever virus Gn protein by a human neutralizing monoclonal antibody with a kappa light chain
Source: PLoS Pathog. 2026 Feb 17;22(2):e1013926. doi: 10.1371/journal.ppat.1013926 (PMC12912543; doi:10.1371/journal.ppat.1013926)
Supplement: S5 Fig — The alignment was generated using ESPript [58]. The GnH sequence of the ZH-501 strain is identical to that of the KEN07-KLF112 strain used for X-ray crystallography. Secondary structure elements are given above the alignment, the numbers in green denote positions of cysteines forming disulphide bonds. The boxes underneath the alignment denote amino-acids interacting with RVFV-379, and are colored black or grey, for heavy or light chain contacts, respectively. (DOCX) [file ppat.1013926.s006.docx]

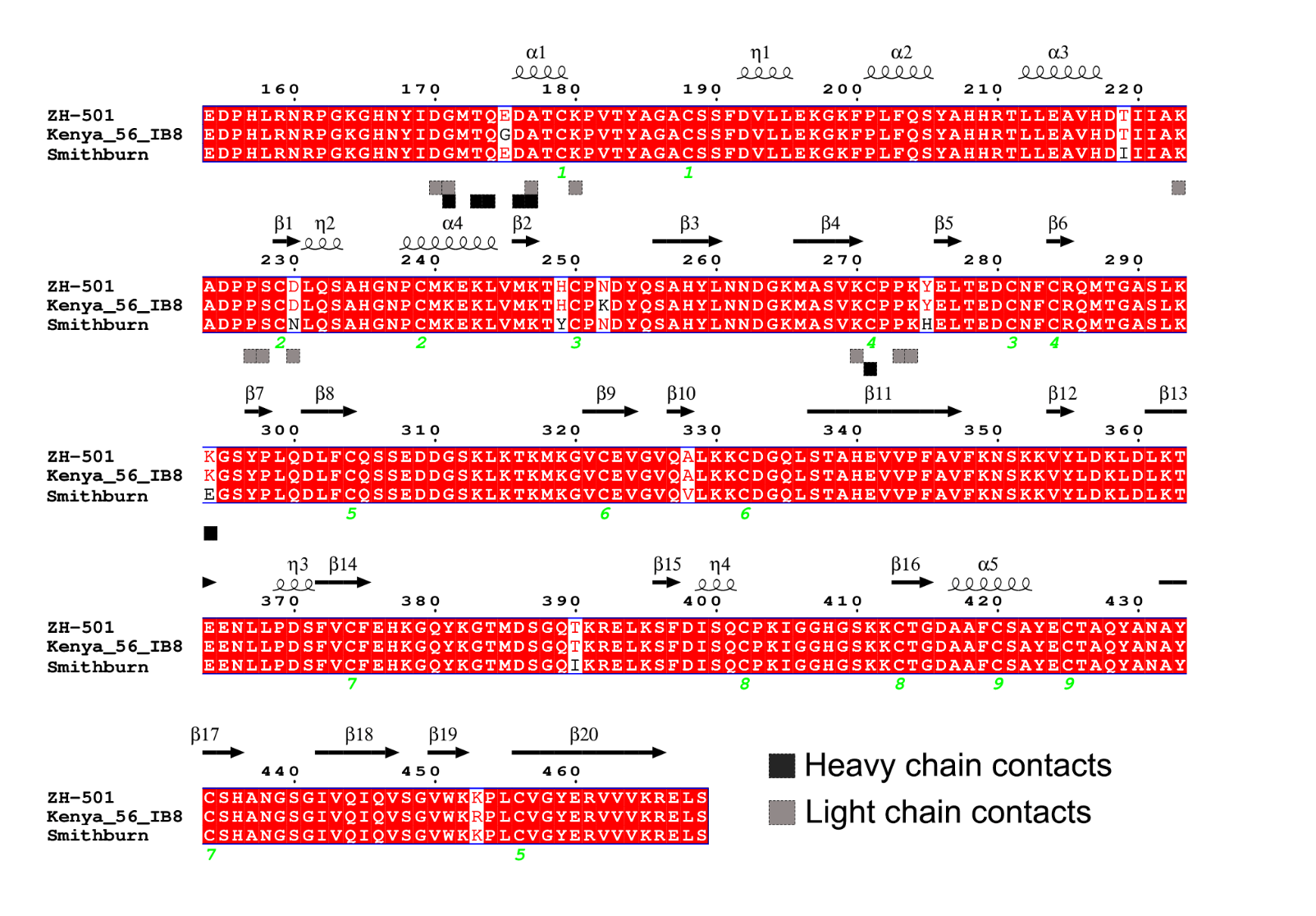


**S5 Fig. Alignment of Gn^H^ proteins of RVFV strains used to test neutralization sensitivity of antibodies.** The alignment was generated using ESPript (6). The Gn^H^ sequence of the ZH-501 strain is identical to that of the KEN07-KLF112 strain used for X-ray crystallography. Secondary structure elements are given above the alignment, the numbers in green denote positions of cysteines forming disulphide bonds. The boxes underneath the alignment denote amino-acids interacting with RVFV-379, and are colored black or grey, for heavy or light chain contacts, respectively.
