# Supplementary material for: Structural basis for recognition of Rift Valley fever virus Gn protein by a human neutralizing monoclonal antibody with a kappa light chain
Source: PLoS Pathog. 2026 Feb 17;22(2):e1013926. doi: 10.1371/journal.ppat.1013926 (PMC12912543; doi:10.1371/journal.ppat.1013926)
Supplement: S6 Fig — Viral titer data were obtained using an infectious cell culture assay in technical triplicate to assess the efficacy of mAbs in the therapeutic setting. Viral cytopathic effect was used to calculate 50% endpoint values. Lower limits of detection (LOD) were 1.49 log10 50% cell culture infectious dose (CCID50)/mL for serum or 2.1 log10 CCID50/g tissue. In samples presenting with virus below the limit of detection (LOD), the representative value of LOD was assigned for analysis. Human mAb DENV 2D22 (specific to an unrelated target, dengue virus) was used as the isotype-matched negative control mAb. Four animals per group were sacrificed on 3 d.p.i. for analysis of virus in serum (A), liver (B), and spleen (C). The dotted line represents the LOD. Data were analyzed using an ordinary one-way ANOVA correcting for multiple comparisons using a Dunnett’s post-test to compare the differences in viral titer (**P < 0.01, *P < 0.05). (DOCX) [file ppat.1013926.s007.docx]

**S6 Fig. Treatment with mAb RVFV-379 reduced viral titers in various organs at low doses.** Viral titer data were obtained using an infectious cell culture assay in technical triplicate to assess the efficacy of mAbs in the therapeutic setting. Viral cytopathic effect was used to calculate 50% endpoint values. Lower limits of detection (LOD) were 1.49 log_10_ 50% cell culture infectious dose (CCID_50_)/mL for serum or 2.1 log_10_ CCID_50_/g tissue. In samples presenting with virus below the limit of detection (LOD), the representative value of LOD was assigned for analysis. Human mAb DENV 2D22 (specific to an unrelated target, dengue virus) was used as the isotype-matched negative control mAb. Four animals per group were sacrificed on 3 d.p.i. for analysis of virus in serum (A), liver (B), and spleen (C). The dotted line represents the LOD. Data were analyzed using an ordinary one-way ANOVA correcting for multiple comparisons using a Dunnett’s post-test to compare the differences in viral titer (***P*<0.01, **P*<0.05).
