# Supplementary material for: Structural basis for recognition of Rift Valley fever virus Gn protein by a human neutralizing monoclonal antibody with a kappa light chain
Source: PLoS Pathog. 2026 Feb 17;22(2):e1013926. doi: 10.1371/journal.ppat.1013926 (PMC12912543; doi:10.1371/journal.ppat.1013926)
Supplement: S1 Table — (DOCX) [file ppat.1013926.s008.docx]

**S1 Table. Data collection and refinement statistics.**

**Beamline** DLS I24

**Wavelength** (Å) 0.6199

**Space Group** *C*222_1_

**Cell dimensions**

a b c (Å) 118.09 187.37 157.88

𝛼 β 𝛾 (°) 90.00 90.00 90.00

**Resolution range** (Å) 39.47-2.09 [2.14-2.09]

**Rmerge**  0.274 [>1]

**I/σ (I)** 8.6 [0.9]

**CC1/2** 0.997 [0.326]

**Completeness** (%) 99.94 [99.94]

**Multiplicity** 13.9 [14.1]

**Refinement Statistics**

**Resolution** (Å) 39.47-2.09

**No. reflections** 103,248

**Rwork/Rfree** 0.212/0.251

**No. atoms**

All 11,339

Ligand 43

Water 540

**Average B-factors**

Protein 47.6

Ligand 42.8

Water 43.8

**Ramachandran** (%)

Favored 97.64

Allowed 2.36

Outlier 0.00

**Root mean square deviations**

Bond lengths (Å) 0.003

Bond angles (°) 0.73

**MolProbity score** 1.15

The value for the highest-resolution shell is shown between square brackets.
