# Supplementary material for: Structural basis for recognition of Rift Valley fever virus Gn protein by a human neutralizing monoclonal antibody with a kappa light chain
Source: PLoS Pathog. 2026 Feb 17;22(2):e1013926. doi: 10.1371/journal.ppat.1013926 (PMC12912543; doi:10.1371/journal.ppat.1013926)
Supplement: S2 Table — (DOCX) [file ppat.1013926.s009.docx]

**S2 Table. Natural genetic variation and respective frequency of polymorphisms in RVFV Gn^H^ in close proximity of the RVFV-379 paratope of 298 full-length M segments of RVFV.**

| **RVFV Gn residue** | **Strains with denoted mutations**  Mutation: strain name (accession number) (c) =complete genome available (strain host species origin) | **Frequency within complete M segment sequences: (n=298) (%)** |
| --- | --- | --- |
| **Ile169** | none | - |
| **Asp170** | none | - |
| **Gly171** | none | - |
| **Met172** | **M172R**: KEN/Bar-035/07 (HM586969.1) (c) (human)  **M172R**: KEN/Bar-032/07 (HM586968.1) (c) (human)  **M172R**: KEN/Mal-032/07 (HM586967.1) (c) (human)  **M172V**: Beijing-01 (KX611606.1) (c)(human) | M172R: 3/298 (1.01%)  M172V: 1/298 (0.34%) |
| **Thr173** | **T173L**: KEN/Bar-035/07 (HM586969.1) (c) (human)  **T173L**: KEN/Bar-032/07 (HM586968.1) (c) (human)  **T173L**: KEN/Mal-032/07 (HM586967.1) (c) (human) | T173L: 3/298 (1.01%) |
| **Gln174** | none | - |
| **Glu175** | **E175G**: Kenya_56_(IB8) (OQ440151.1) (c) (cow)  **E175G**: Kenya_56_(IB8) (DQ380190.1) (c) (cow) | E175G: 2/298 (0.67%) |
| **Asp176** | none | - |
| **Ala177** | **A177T**: SA1797/10 (MG659803.1) (c) (human)  **A177T**: SA1796/10 (KY126700.1) (c) (human) | A177T: 2/298 (0.67%) |
| **Thr178** | none | - |
| **Lys180** | none | - |
| **Lys223** | none | - |
| **Asp225** | none | - |
| **Pro226** | none | - |
| **Pro227** | none | - |
| **Ser228** | none | - |
| **Cys229** | none | - |
| **Asp230** | **D230G**: M1975/Bov (KX944832.1) (c) (cow)  **D230N**: Smithburn (DQ380193.1) (c) (host not specified)  **D230N**: RSA/OBP/RVFVSmithburn/LAV_isolate (OP146108.1) (c) (host not specified) | D230G: 1/298 (0.34%)  D230N: 2/298 (0.67%) |
| **Gln255** | none | - |
| **Ser256** | none | - |
| **Ser268** | none | - |
| **Lys270** | **K270X**: 2021001358_M\|Uganda (ON060837.1) (incomplete genome) (human) | K270X: truncation 1/298 (0.34%) |
| **Cys271** | **C271X**: 2021001358_M\|Uganda (ON060837.1) (incomplete genome) (human) | C271X: truncation 1/298 (0.34%) |
| **Pro272** | **P272X**: 2021001358_M\|Uganda (ON060837.1) (incomplete genome) (human) | P272X: truncation 1/298 (0.34%) |
| **Pro273** | **P273X**: 2021001358_M\|Uganda (ON060837.1) (incomplete genome) (human) | P273X: truncation 1/298 (0.34%) |
| **Lys274** | **K274X**: 2021001358_M\|Uganda (ON060837.1) (incomplete genome) (human) | K274X: truncation 1/298 (0.34%) |
| **Leu292** | none | - |
| **Lys293** | none | - |
| **Lys294** | **K294E**: Smithburn (DQ380193.1) (c) (host not specified) | K294E: 1/298 (0.34%) |
