# Supplementary material for: Structural basis for recognition of Rift Valley fever virus Gn protein by a human neutralizing monoclonal antibody with a kappa light chain
Source: PLoS Pathog. 2026 Feb 17;22(2):e1013926. doi: 10.1371/journal.ppat.1013926 (PMC12912543; doi:10.1371/journal.ppat.1013926)
Supplement: S3 Table — Empty cells indicate that no contact was observed between RVFV-379 and GnH. (DOCX) [file ppat.1013926.s010.docx]

**S3 Table.** **Residues on the surface of Gn^H^ within 5Å of a residue on RVFV-379 heavy (H) or light (L) and their contact type**. Empty cells indicate that no contact was observed between RVFV-379 and Gn^H^.

| **Amino acid and position** | **Heavy chain** | **Light chain** |
| --- | --- | --- |
| **Ile169** |  |  |
| **Asp170** |  | **hydrophobic interaction** with Tyr92  connects via **water bridge** with Tyr92  connects via **water bridge** with Thr93 |
| **Gly171** | **hydrogen bond** with Ser99  **hydrogen bond** with Tyr98 | **hydrogen bond** with Ser91  **hydrogen bond** with Tyr92  **hydrophobic interaction** with Phe32  connects via **water bridge** with Thr93  connects via **water bridge** with Tyr92 |
| **Met172** |  |  |
| **Thr173** | **3 hydrogen bonds** with Arg50 |  |
| **Gln174** | **hydrophobic bond** with Ser99  **hydrophobic bond** with Tyr98  **hydrogen bond** with Glu95  **hydrogen bond** with Ala100  **hydrogen bond** with Ser99  **hydrogen bond** with Ala100  **hydrogen bond** with Arg50  connects via a **water bridge** with Glu95 |  |
| **Glu175** |  |  |
| **Asp176** | **hydrogen bond** with Tyr33  connects via a **water bridge** with Ser56  forms a **salt bridge** with Arg 50 |  |
| **Ala177** | **hydrogen bond** with Tyr33 | connects via a **water bridge** with Thr93 |
| **Thr178** |  |  |
| **Lys180** |  | connects via a **water bridge** to Gln27 |
| **Lys223** |  | **hydrogen bond** with Tyr92  connects via a **water bridge** to Gln27 |
| **Asp225** |  |  |
| **Pro226** |  |  |
| **Pro227** |  | **hydrophobic interaction** with atom CG of Glu30 |
| **Ser228** |  | **hydrogen bond** with Glu30  connects via a **water bridge** with Glu30 |
| **Cys229** |  |  |
| **Asp230** |  | **hydrophobic bond** with Phe67 |
| **Gln255** |  |  |
| **Ser256** |  |  |
| **Ser268** |  |  |
| **Lys270** |  | **hydrophobic interaction** with Phe32  **hydrogen bond** with Glu30 |
| **Cys271** | **hydrogen bond** with Tyr98 |  |
| **Pro272** |  |  |
| **Pro273** |  | **hydrophobic interaction** with Tyr49  **hydrophobic interaction** with Ile50 |
| **Lys274** |  | **hydrogen bond** with Tyr49 |
| **Leu292** |  |  |
| **Lys293** |  |  |
| **Lys294** | **hydrogen bond** with Asp31B |  |
